# PHOENIX: a study protocol for a pilot randomised controlled trial of pre-emptive pharmacogenomic panel testing in acute care settings with health economic evaluations

**DOI:** 10.1136/bmjopen-2026-121606

**Published:** 2026-10-05

**Authors:** Stefanie Lip, Pieter Theo Pepler, Alex McConnachie, Leslie J Sheffield, Sam Mostafa, Linsay McCallum, Neil Hawkins, Olivia Wu, Louise Bennett, Iain Frater, Katriona Brooksbank, Sean Macbride-Stewart, Victoria M Pratt, Brandon Franklin, Allan Sheffield, Sandosh Padmanabhan

**Affiliations:** 1 School of Cardiovascular and Metabolic Health, University of Glasgow, Glasgow, Scotland, UK; 2 School of Health and Wellbeing, University of Glasgow, Glasgow, Scotland, UK; 3 MyDNA, GenebyGene, Melbourne, Victoria, Australia; 4 Centre for Medicine Use and Safety, Monash University, Melbourne, Victoria, Australia; 5 MVLS Innovation Engagement and Enterprise, University of Glasgow, Glasgow, UK; 6 West of Scotland Innovation Hub, NHSGGC, Glasgow, Scotland, UK; 7 Pharmacy Services, NHSGGC, Glasgow, Scotland, UK; 8 Agena Bioscience Inc, San Diego, California, USA

**Keywords:** CLINICAL PHARMACOLOGY, GENETICS, Randomized Controlled Trial, HEALTH ECONOMICS

## Abstract

**Introduction:**

Pharmacogenomic (PGx) panel testing may reduce adverse drug reactions (ADRs), treatment failures and downstream healthcare use by aligning prescribing with germline genetic variation. The PREPARE trial reported an approximately 30% reduction in clinically relevant ADRs using a pre-emptive multigene panel, but its open-label design, patient-reported endpoint and subsequent methodological critiques limit its use for National Health Service (NHS) decision-making. Whether panel-based PGx delivers benefit in acute NHS secondary care, where prescribing is time-critical, patients are older and multimorbid and outcomes can be linkage-adjudicated, remains unknown.

**Methods and analysis:**

PHOENIX is a pragmatic, pilot, parallel-group, individually randomised controlled trial with blinded outcome adjudication, in NHS Greater Glasgow and Clyde (NHSGGC) hospitals and NHS Golden Jubilee National Hospital. Adults newly prescribed an eligible PGx-relevant index drug are identified through a data-driven screening system integrating electronic prescribing, community prescribing linkage and record review and randomised 1:1 to PGx-guided care or standard care with delayed PGx testing. In the intervention arm, buccal-swab DNA is genotyped (Clinical Laboratory Improvement Amendments/College of American Pathologist (CLIA/CAP)-accredited laboratory) and a Clinical Pharmacogenetics Implementation Consortium (CPIC)/Dutch Pharmacogenetics Working Group (DPWG)-based report returned within approximately 10 days; results are research-use-only. The trial aims to recruit 2000–4000 participants constrained by a fixed operational end-date (30 September 2026). The primary outcome is index-drug-related ADR or treatment failure within 3 months, adjudicated using Common Terminology Criteria for Adverse Events (CTCAE) V.6 and validated causality tools, with deterministic linkage via the West of Scotland Safe Haven. Secondary outcomes include ADR severity, hospitalisations, mortality, actionable prescribing uptake, biomarker levels, EuroQol 5-Dimension 5-Level questionnaire (EQ-5D-5L) and healthcare utilisation. The primary analysis is intention-to-treat logistic regression adjusted for stratification factors; a within-trial cost-utility analysis estimates cost per quality-adjusted life year (QALY).

**Ethics and dissemination:**

Approved by Scotland A Research Ethics Committee (24/SS/0064; IRAS 344412; NHS R&I INGN24MG113). Co-sponsors are NHSGGC and the University of Glasgow. Findings will be disseminated via peer-reviewed publication, conferences, patient and public involvement and engagement-co-produced lay summaries and the trial website.

**Trial registration number:**

NCT06907784.

**STRENGTHS AND LIMITATIONS OF THIS STUDY**

- Individual randomisation with blinded outcome adjudication and deterministic linkage to comprehensive National Health Service (NHS) datasets, addressing the principal methodological limitations of prior open-label pharmacogenomic implementation trials.
- Evaluation of a multigene panel across 74 medicines and multiple specialties, with an integrated within-trial health-economic evaluation generating NHS-specific parameters.
- A novel data-driven screening system integrating hospital and community prescribing data enables population-level identification of eligible patients in near real-time.
- As a pilot with a fixed, funding-determined end-date, the trial is not powered for definitive drug-specific or gene-specific effects; its purpose is to generate design parameters for a definitive trial.
- Genotyping uses an external accredited laboratory and results are research-use-only, so the trial evaluates the index treatment episode rather than lifelong pre-emptive prescribing; the population has limited ethnic diversity.

## Introduction

### Background and rationale

 Over the last decade, pharmacogenomics has moved from concept to policy priority within the UK, but implementation in the UK National Health Service (NHS) remains fragmented and largely limited to a small number of single gene-drug pairs. The NHS England Accelerating Genomic Medicine strategy^1^ and the joint Royal College of Physicians/British Pharmacological Society Personalised Prescribing report^2^ explicitly call for transition from indication-specific single-gene testing to pre-emptive, panel-based pharmacogenomic (PGx) services with data re-used across primary, secondary and tertiary care.^3^ This direction has been strengthened by the Network of Excellence for Pharmacogenomics and Medicines Optimisation^1^ and implementation programmes such as PROGRESS to pilot panel-based testing and clinical decision support in routine practice.^3^ These developments create strong momentum for adoption, but also increase the need for robust, NHS-specific evidence, demonstrating clinical benefit, operational feasibility, cost-effectiveness and equity of impact.

Current NHS implementation is dominated by single gene-drug applications deployed in high-risk niches (eg, HLA-B*57:01 for abacavir, *DPYD* for fluoropyrimidines, *MT-RNR1* for neonatal aminoglycoside ototoxicity). Recently, National Institute for Health and Care Excellence (NICE) diagnostics guidance DG59^4^ concluded that *CYP2C19* genotyping to guide clopidogrel use after stroke/transient ischaemic attack is clinically effective and cost-saving, but national implementation would require ~100 000 tests per year—a >30% increase in NHS genomic laboratory capacity—at turnaround times compatible with urgent stroke pathways.^5^ Adoption has consequently been limited, with unresolved questions about equitable access and service sustainability. At the same time, population studies show that most adults will over time be prescribed at least one medication with guideline-based PGx recommendations, and many, particularly older adults with multimorbidity and polypharmacy, will receive several.^4 6^ Panel-based testing, where common pharmacogenes are assayed once and results reused, offers economies of scale, harmonised reporting and better alignment with real prescribing than sequential single-gene commissioning.^3^

The PREPARE trial provides the strongest current evidence supporting this model.^7^ Using a 12-gene panel across seven European countries in an open-label, cluster-randomised, crossover design, PREPARE reported an approximately 30% reduction in clinically relevant adverse drug reactions (ADRs) over 12 weeks among patients with actionable gene-drug pairs, with high uptake of Dutch Pharmacogenetics Working Group (DPWG) recommendations.^7^ However, its primary endpoint relied on short-term, patient-reported ADRs; it did not systematically assess effectiveness, health-service utilisation or longer-term outcomes; and ADR reductions were also observed among patients without actionable variants.^8^ Subsequent correspondence raised concerns regarding differential loss to follow-up between arms, biased ascertainment given unblinded patient-reported outcome collection and limited inter-rater agreement on causality in an independently reassessed subsample.^9–12^ In a post hoc analysis adjusting for confounding, the whole-population effect attenuated from a 30% to a 13% reduction and was no longer statistically significant, while the actionable-genotype effect increased to 39%.^11^ The magnitude and robustness of benefit, in particular its expression in objectively ascertained clinical and economic endpoints, therefore remain insufficiently characterised to support NHS-scale implementation decisions.

The NHS is developing a distinct PGx model with nationally coordinated governance, secure data environments and vendor-neutral integration into electronic prescribing.^13^ The clinical effectiveness, operational performance and economic value of a panel-based PGx service embedded within this architecture, particularly in the high-volume context of acute care, are not yet known. Existing economic evaluations suggest potential cost-effectiveness for single-gene and multigene approaches,^14 15^ but lack NHS-specific effect sizes, utilisation patterns and distributional impacts.

One might argue that single-gene evidence, international panel data and modelling suffice for national implementation with observational evaluation, mirroring rare-disease and cancer genomics.^16^ But PGx is potentially universal, and widespread implementation requires high-throughput testing, rapid turnaround and sophisticated decision support carrying substantial cost and operational implications,^3 17^ justifying a higher evidentiary threshold before transition to panel-based, lifetime PGx records.

PHOENIX is designed to address this gap. It is not intended to replicate PREPARE, nor to test whether its effect estimate generalises to a UK population. Rather, it generates an independent, NHS-specific causal estimate using a design that directly addresses the principal methodological limitations of the existing evidence base: individual rather than cluster randomisation; blinded adjudication of clinically defined outcomes rather than unblinded patient-reported symptoms; linkage-based ascertainment of objective endpoints including hospitalisation, treatment failure and mortality; and a prospective within-trial economic evaluation. The question PHOENIX addresses is not whether an effect observed elsewhere applies here, but whether a clinically and economically meaningful effect is demonstrable when panel-based PGx prescribing is evaluated under these conditions within a unified NHS system. These design considerations align with lessons subsequently drawn by the PREPARE investigators regarding endpoint selection, blinding, randomisation, crossover design and analysis.^8^

Scotland, and NHS Greater Glasgow and Clyde (NHSGGC) in particular, provide a uniquely advantageous setting. NHSGGC serves approximately 1.2 million people, uses a single Community Health Index (CHI) number for deterministic linkage of hospital, prescribing and mortality records and benefits from near-universal inpatient hospital electronic prescribing and a mature, accredited Safe Haven for secure data analytics. The absence of prescription charges reduces socioeconomic confounding in medication uptake. To our knowledge, no previous study has evaluated a multigene PGx panel using individual randomisation, blinded adjudication and linked NHS routine data in this setting.

### Objectives

The research question, structured using the PICOT format, is presented in table 1.

**Table 1:** Research question in PICOT format

Participants	Adults (≥18 years) admitted to hospital wards or attending specialist outpatient clinics who are newly prescribed a medication with known pharmacogenomic implications and are capable of providing informed consent (or via a legal representative/welfare guardian/next of kin), and able to provide a buccal swab for PGx testing.
Intervention	PGx testing of DNA from a buccal swab; results returned within 7–10 days to the treating clinician to inform medicine selection and dosing, in addition to standard clinical care.
Comparator	Standard care arm where PGx testing is performed but results are delayed (tested and returned after ~3 months), with usual clinical management in the interim.
Primary Outcome	Composite of adverse drug reactions (CTCAE ≥2) and/or treatment failures (applies to specific drugs where treatment failure is captured by hospital admissions with the condition for which the index drug was commenced).
Time point of main interest	Follow-up for at least 12 weeks post-randomisation with additional long-term data collection (eg, hospital admissions, medication changes) through linked health records up to study completion (~September 2026).

Common Terminology Criteria for Adverse Events (CTCAE)

PGx, pharmacogenomic.

The PHOENIX pilot trial aims to: (1) estimate the preliminary effectiveness of PGx panel-guided prescribing in reducing index-drug-related ADRs and treatment failure over 3 months; (2) assess feasibility and implementation of delivering PGx-guided prescribing using an external accredited laboratory, including screening, recruitment, swab logistics, turnaround, clinician uptake and operational barriers; (3) conduct an integrated within-trial economic evaluation and generate parameters for a definitive trial; and (4) explore differential effects across key subgroups.

#### Hypotheses

PHOENIX is estimation-focused and feasibility-focused but underpinned by prespecified hypotheses.

##### Primary hypothesis

Among adults newly prescribed an eligible PGx-relevant index drug in acute secondary care, PGx panel-guided prescribing reduces the composite of index-drug-related ADRs and treatment failure at 3 months versus standard care with delayed PGx testing. This hypothesis is tested using logistic regression adjusted for the randomisation stratification factors under the intention-to-treat principle, with the complier-average causal effect (CACE) and per-protocol analyses as secondary estimators.

##### Secondary hypotheses

Relative to standard care, PGx-guided prescribing (1) reduces ADR severity, (2) reduces hospitalisations, (3) increases guideline-concordant (actionable) prescribing and (4) is associated with favourable short-term cost-utility, each secondary hypothesis is tested using the corresponding model described in the Statistical analysis and Economic evaluation sections. Because the trial is powered for parameter estimation rather than definitive confirmation, hypothesis tests generate NHS-specific design parameters and directional evidence for a future definitive trial.

## Methods and analysis

### Trial design

PHOENIX (NCT06907784) is a pragmatic, pilot, individually randomised, parallel-group controlled trial of multigene PGx panel-guided prescribing in acute secondary care within NHSGGC. It is designated a pilot because all recruitment, follow-up and genotyping must conclude by 30 September 2026, the end date of Living Laboratory funding (online supplemental table 1: SA01). This fixed endpoint constrains the maximum achievable sample size; the trial aims to enrol 2000–4000 participants, allowing estimation of event rates, implementation metrics and economic parameters for a definitive trial. The trial is embedded within routine NHS workflows while maintaining internal validity through individual randomisation, prespecified stratification and blinded outcome adjudication, adhering to Good Clinical Practice (GCP) and Standard Protocol Items: Recommendations for Interventional Trials (SPIRIT) 2025.^18^

The panel is pre-emptive in that a single assay produces results that are, in principle, lifelong and reusable. Within this trial, however, testing is triggered reactively by an eligible index prescription, and results are applied only within the trial follow-up period and not stored in the NHS record. The registered title’s use of ‘pre-emptive’ therefore refers to the panel-based, reusable nature of the assay (‘reactive testing with planned re-use’) rather than to testing performed in advance of prescribing need. All substantial and non-substantial protocol amendments including rationale are listed in online supplemental table 1.

### Implementation framework

The feasibility and implementation evaluation is structured using established implementation-science frameworks. Implementation metrics specified in the protocol map onto the implementation-outcomes taxonomy of Proctor *et al*^19^—acceptability and adoption (clinician uptake of actionable recommendations), feasibility (screening yield, buccal-swab logistics and shipment), fidelity (turnaround time and whether reports were applied as intended) and penetration (reach across specialties and index-drug groups). The Reach, Effectiveness, Adoption, Implementation and Maintenance (RE-AIM) framework^20^ informs reporting of the recruitment pipeline, clinician adoption and the potential for sustained NHS delivery.

### Study setting

The trial is conducted across all inpatient medical, surgical and specialist secondary-care wards and outpatient clinics of NHSGGC Acute Services, with expansion of recruitment to include NHSGGC Mental Health Services (online supplemental table 1: SA02) and the NHS Golden Jubilee Hospital (online supplemental table 1: NSA07). Within NHSGGC, inpatient prescribing is supported by HEPMA and a single CHI number, enabling deterministic linkage and outcome capture. The West of Scotland Safe Haven is an accredited Trusted Research Environment enabling secure, governance-approved linkage of routinely collected NHS data—hospital admissions, community dispensing and mortality records—using the CHI number, without researchers accessing identifiable data directly. It provides the data-linkage backbone for outcome ascertainment. No investigational medicinal products are introduced.

### Eligibility criteria

Full inclusion and exclusion criteria, mapped to the current protocol (V.3.2) with amendment numbers, are provided in online supplemental table 2. In brief, participants are aged ≥18 years, able to consent directly or via a legal representative, and newly prescribed an index drug—defined as no prescription for the same drug within the preceding 3 months (reduced from 12 months by amendment SA01), confirmed via hospital and community prescribing records. The index-drug list comprises 74 medications whose actionable drug-gene interactions map to 15 pharmacogenes; a further gene, CYP1A2, is genotyped to support phenoconversion analyses but does not itself confer eligibility. Drugs and genes were selected on the basis of Clinical Pharmacogenetics Implementation Consortium (CPIC)/Dutch Pharmacogenetics Working Group (DPWG) guidance, relevance to acute and specialist prescribing, expected frequency and likelihood of influencing prescribing during follow-up. The full drug and gene/variant lists are provided in online supplemental tables 3 and 4.

For inpatients, an index drug is defined by an active Hospital Electronic Prescribing and Medicines Administration (HEPMA) prescription that has proceeded to administration; the majority are therefore dispensed and administered rather than merely written. For outpatients, enrolment occurs only after the participant confirms they have received and commenced the medication (SA02), avoiding enrolment of undispensed prescriptions. Where more than one eligible index drug is newly prescribed during the same admission or eligibility window, the less frequently prescribed (rarer) drug is designated the primary index drug and determines the stratification group and primary-outcome denominator; this prioritises representation of less common drug-gene pairs given the drug caps applied to high-frequency drugs. PGx recommendations in the intervention arm nonetheless apply to all index and clinically relevant concomitant medications.

### Screening and recruitment

Screening is conducted daily using a data-driven workflow integrating routine NHS data sources within the West of Scotland Safe Haven. An automated system interrogates HEPMA to generate real-time alerts for newly prescribed index drugs, enhanced by deterministic CHI-based linkage to community prescribing data to confirm genuine treatment initiation and exclude recent prior exposure. Scottish community prescribing records capture over 98% of NHS-funded dispensed prescriptions, and the absence of prescription charges supports comprehensive ascertainment. Potential participants identified through automated screening are reviewed manually by the research team to confirm eligibility and verify suitability with the treating teams before approach. At NHS Golden Jubilee, which operates outside HEPMA, screening is by manual record review (NSA07); for outpatient clinics, screening follows liaison with the responsible consultant (SA02).

Screening logs record all individuals identified, approached, enrolled or not enrolled (with reasons), providing a complete audit trail. These will be summarised in a Consolidated Standards of Reporting Trials (CONSORT)-style flow diagram and used to characterise potential recruitment bias by comparing eligible non-participants with enrolled participants, where governance permits. Recruitment continues until the operational end-date or until ~4000 participants.

### Consent

Informed consent is obtained by trained research nurses or doctors, using short and full participant information sheets, in accordance with GCP. For participants lacking capacity, consent is sought from an appropriate legal representative under the Adults with Incapacity (Scotland) Act 2000. A unique trial identifier is recorded in the electronic case report form; the CHI number is stored separately in a secure linkage file.

### Randomisation and blinding

Participants are randomised 1:1 via CASTOR Electronic Data Capture using variable permuted blocks (sizes 2–4) stratified by specialty and index-drug group, with allocation concealed until assignment. Participants and treating clinicians remain blinded unless the PGx report contains an actionable recommendation requiring a medication change; any necessary change creates unavoidable functional unblinding, minimised by limiting disclosure. Outcome adjudication is blind to allocation.

### Interventions

At enrolment, all participants provide a buccal swab (fasting ≥1 hour). In the intervention arm, the sample is shipped to a Clinical Laboratory Improvement Amendments/College of American Pathologist (CLIA/CAP)-accredited laboratory (Gene by Gene, Houston, USA) for genotyping, and myDNA (Melbourne, Australia; UKCA class I device software) generates a structured CPIC/DPWG-based report covering the index drug and concomitant medications, returned to the treating clinician typically within approximately 10 days. Because the assay is not Conformité Européenne (European Conformity)/UK Conformity Assessed (CE/UKCA)**-**marked for NHS clinical use, results are research-use-only, accessible only to authorised trial clinicians, applied solely within the trial episode and not uploaded to NHS records. The treating consultant retains responsibility for all prescribing; changes are not automatic. An actionable recommendation is one that, per CPIC/DPWG guidance, warrants a change of drug or dose or added monitoring (eg, an alternative antiplatelet in a CYP2C19 poor metaboliser on clopidogrel; alternative analgesia in a CYP2D6 poor or ultrarapid metaboliser on codeine or tramadol). Full laboratory methods, report generation and the procedure on receipt (including handling of failed samples and discharged patients) are provided in online supplemental methods 1.

The intended end-to-end turnaround is approximately 7–10 days; because samples are externally processed, observed turnaround varies and will be reported descriptively (median and IQR, overall and by assay version) as an implementation outcome. Turnaround does not contribute to the primary analysis population; intention-to-treat includes all randomised participants irrespective of when their report was returned. ADRs or treatment failures occurring before report return are captured and adjudicated identically; the proportion of primary events occurring before report availability is a prespecified implementation metric quantifying clinical opportunity potentially lost to turnaround.

In the comparator arm, participants receive usual care without access to PGx results for 3 months. At 3 months, stored DNA is analysed using the same panel and a report reflecting current prescriptions is returned to the treating consultant; recommendations are not returned for baseline medications discontinued in the interim. Multigene panel testing is not part of routine standard care at any participating site, current NHS Scotland practice is limited to a few nationally commissioned single gene-drug applications, so the comparator reflects genuine standard care. Contamination is minimised by design: the assay is external and research-use-only, reports are never uploaded and any routine single-gene test relates to a specific pathway captured in linked data. Although PGx results are not returned in this arm during follow-up, genotyping of the stored sample provides a complete profile for every participant. Because genotype is fixed and the index drug recorded at randomisation, index-drug actionability at randomisation can be derived identically in both arms, independently of the returned report, supporting a prespecified analysis of the intervention effect within the actionable-genotype subgroup in whom any effect is expected to concentrate.

To limit disproportionate recruitment of the most common PGx-relevant drugs (eg, clopidogrel), a flexible drug cap may be applied, reviewed by the Trial Steering Committee (TSC) considering scientific validity, prescribing patterns, feasibility and the fixed timeline. All drugs are licensed products used within usual indications; no investigational medicinal products are introduced.

### Participant timeline and follow-up

Follow-up occurs for 3 months post-randomisation with monthly participant-reported outcomes, review of medication changes and a protocolised 4-week blood test for drugs with monitoring requirements (both arms). The schedule of enrolment, interventions and assessments is shown in figure 1: Schedule of enrolment, interventions and assessments and online supplemental table 5. Adherence to the index and concomitant drugs is assessed monthly by a validated self-report measure, supplemented by CHI-linked community dispensing (proportion-of-days-covered), summarised descriptively by arm and incorporated into the CACE analysis.

**Figure 1:**
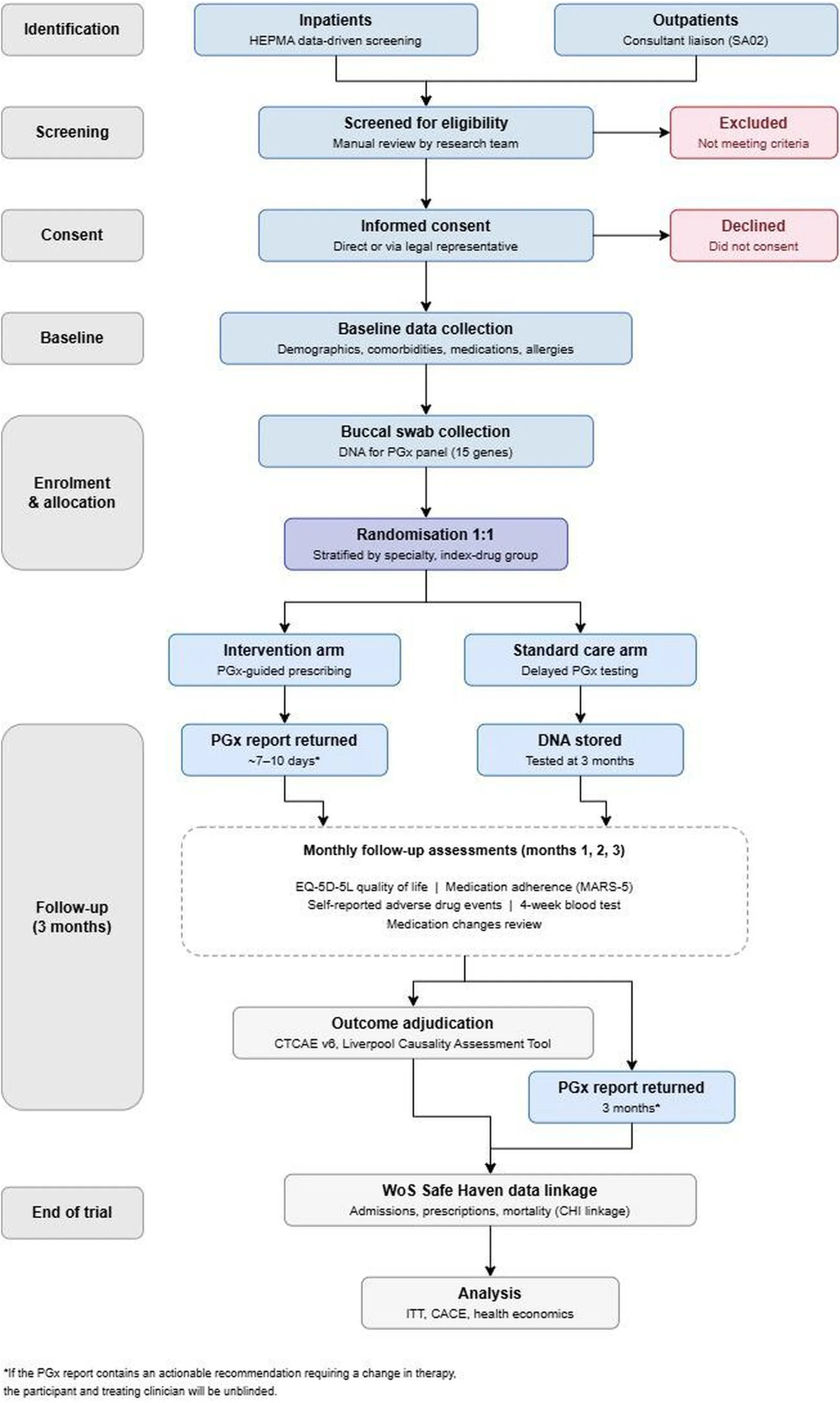
Schedule of enrolment, interventions and assessments. CACE, complier-average causal effect; CTCAE, Common Terminology Criteria for Adverse Events; CHI, Community Health Index; EQ-5D-5L, EuroQol 5-Dimension 5-Level questionnaire; ITT, Intention-to-treat; MARS-5, Medication Adherence Report Scale; PGx, pharmacogenomic.

### Outcomes

The primary outcome is an index-drug-related ADR or treatment failure within 3 months. ADR attribution is by a blinded adjudication panel using validated causality tools (Liverpool Causality Assessment Tool or WHO-UMC, by event type), weighing temporal relationship, dechallenge/rechallenge, alternative causes and biological plausibility; only events at least possibly related are counted. Treatment failure is defined per drug class as failure of the index drug to achieve its intended effect (eg, a recurrent vascular event on clopidogrel; admission or escalation for the treated condition; or discontinuation/switching for lack of efficacy), operationalised through linked data and confirmed by the panel; full mapping is prespecified in the Statistical Analysis Plan (SAP). Events are graded using CTCAE V.6.

Secondary outcomes include ADR severity (CTCAE V.6); all-cause and cause-specific hospitalisations; all-cause mortality; EuroQol 5-Dimension 5-Level questionnaire (EQ-5D-5L); participant-reported adverse effects; proportion and timing of PGx recommendation uptake; drug-specific biomarker levels at ~4 weeks; and healthcare utilisation via linked data (online supplemental methods S4). Implementation measures include turnaround time, proportion of actionable reports, clinician uptake and the proportion of primary events occurring before results were available. Baseline PGx burden (the frequency of actionable drug-gene interactions across the whole prescription at consent) and phenoconversion prevalence are reported as descriptive baseline characterisations (online supplemental methods S2 and S3).

### Statistical analysis

A detailed SAP will be finalised and approved by the TSC before database lock and made publicly available. The primary analysis population will be the full analysis set (all randomised participants meeting the eligibility criteria and providing a buccal swab). The primary endpoint is analysed by logistic regression with fixed effects for the stratification factors and prespecified covariates (age, sex, baseline polypharmacy count, Scottish Indext of Multiple Deprivation (SIMD) quintile), summarised as an adjusted OR with 95% CI. Secondary outcomes are analysed with models appropriate to their distribution (ordinal logistic for severity; negative binomial or Poisson for counts; Cox proportional hazard for time-to-event; linear for continuous outcomes; logistic/linear for uptake and adherence), each adjusted for the same factors, with full specifications in the SAP. A per-protocol analysis (participants receiving their allocated intervention as intended) and a CACE analysis are prespecified secondary estimators. Baseline characteristics, including genotype and predicted-phenotype frequencies, phenoconversion prevalence and whole-prescription actionability, are summarised descriptively by arm to allow assessment of balance, without significance testing of baseline differences. Exploratory subgroup analyses assess effect modification by sex, age, SIMD, ethnicity, polypharmacy, smoking status, phenoconversion status and consent type via prespecified interaction terms; these will not influence the primary analysis. A within-arm exploratory analysis compares intervention-arm outcomes where genotype-predicted and effective phenotypes agree versus diverge. Missing primary-outcome data are expected to be minimal given linkage; complete-case analysis is primary, with multiple imputation as sensitivity analysis. No interim effectiveness analyses or formal stopping rules are planned.

### Economic evaluation

A short-term, within-trial cost-utility analysis over 3 months will estimate direct costs (PGx testing, logistics, healthcare utilisation) and derive Quality-Adjusted Life Years (QALY) from EQ-5D-5L, informing a future definitive cost-effectiveness study and assessment of NHS service delivery models including local versus external laboratory provision.

### Data collection and management

Data are collected via CASTOR EDC (GCP-compliant, 21 CFR Part 11-ready) with real-time validation and audit trails. Identifiable data (CHI) are stored in an encrypted linkage file separate from outcomes; Safe Haven linkage is under established governance. Access is role-restricted.

### Sample size

PHOENIX is a pilot with a planned target of up to 4000 participants (~1000–2000 per arm), the achieved size depending on recruitment and the fixed end-date. It is not based on a single definitive power calculation but on plausible event rates and effect sizes from the literature and PREPARE. Primary event rates are expected at 15–25%. For an OR of ~0.7–0.8, ~1000 per arm gives power typically above 80% for larger effects at higher event rates (one-sided 10%); ~2000 per arm exceeds 90% across broader assumptions (two-sided 5%). Even lower-end recruitment will yield precise estimates of key design parameters for a definitive trial.

### Trial oversight and monitoring

Oversight is provided by TSC, monitoring scientific conduct, recruitment balance, drug-cap decisions and safety. The Data Monitoring Committee function is delegated to the TSC, which reviews unblinded safety data and may recommend modification, continuation or termination of the trial. The TSC has appointed an independent statistician (Professor Andrea Jorgensen, University of Liverpool) to provide independent review of accumulating safety and effectiveness data. The Trial Management Committee is responsible for operational delivery of the trial. Independent monitoring follows a risk-based plan.

### Patient and public involvement and engagement

Patient and public involvement and engagement (PPIE) has been integral from the earliest stages. Lay contributors shaped the trial question, informed outcome selection and reviewed participant information, consent, recruitment materials and questionnaires. They advised on ethical considerations including inclusion of participants lacking capacity, blinding, communication with general practitioners and handling of PGx results after the study and on practical delivery including the 4-week blood test and minimising primary care burden. A full report of the pre-trial PPIE consultation is available on the trial website.

### Ethical and dissemination

The study is approved by Scotland A Research Ethics Committee (REC 24/SS/0064; IRAS 344412), Caldicott Guardian Approval and NHS R&I (INGN24MG113). Inclusion of participants lacking capacity is justified by the high prevalence of multimorbidity and polypharmacy and undertaken via compliant legal-representative procedures. Randomisation to standard care is appropriate because all participants continue to receive usual NHS treatment and PGx testing is an adjunct not part of routine practice. Amendments are approved by co-sponsors, submitted to the Research Ethics Committee (REC) and regulators and summarised in online supplemental table 1. Principal risks relate to the sensitive nature of genetic information, confidentiality and possible anxiety from medication changes, mitigated through clear information, secure data handling and clinician-mediated communication. All data handling complies with General Data Protection Regulation (GDPR) and UK data-protection legislation, with identifiable data minimised and linkage within the Safe Haven.

Dissemination, overseen by the Trial Management Committee (TMC) and TSC, includes a primary publication, secondary analyses, conference presentations and PPIE-led lay summaries, with a final report within 12 months of database lock. Subject to approvals, de-identified data, a data dictionary and statistical code may be shared through controlled-access repositories including Safe Haven-mediated access.

### Trial status

Protocol V.3.2 (19 December 2025) is the current approved version. Recruitment commenced on 9 April 2025 and ends on 30 September 2026, with 3-month follow-up per participant.

## Supplementary material

10.1136/bmjopen-2026-121606 online supplemental file 1
