# Supplementary material for: PHOENIX: a study protocol for a pilot randomised controlled trial of pre-emptive pharmacogenomic panel testing in acute care settings with health economic evaluations
Source: BMJ Open. 2026 Oct 5;16(10):e121606. doi: 10.1136/bmjopen-2026-121606 (PMC13646959; doi:10.1136/bmjopen-2026-121606)
Supplement: Supplementary file 1 [file bmjopen-2026-121606.reviewer_comments.pdf]

PEER REVIEW HISTORY

BMJ Open publishes all reviews undertaken for accepted manuscripts. Reviewers are asked to complete a checklist review form and are provided with free text boxes to elaborate on their assessment. These free text comments are reproduced below.

ARTICLE DETAILS

Title (Provisional)

PHOENIX: A study protocol for a pilot randomised controlled trial of pre-emptive pharmacogenomic panel testing in acute care settings with health economic evaluations.

Authors

Lip, Stefanie; Pepler, Pieter Theo; McConnachie, Alex; Sheffield, Leslie J; Mostafa, Sam; McCallum, Linsay; Hawkins, Neil; Wu, Olivia; Bennett, Louise; Frater, Iain; Brooksbank, Katriona; Macbride-Stewart, Sean; Pratt, Victoria M; Franklin, Brandon; Sheffield, Allan; Padmanabhan, Sandosh

---

VERSION 1 - REVIEW

**Reviewer**	**1**
**Name**	**Rickles, Nathaniel M.**
**Affiliation**	**University of Connecticut School of Pharmacy, Pharmacy Practice**
**Date**	**01-Jul-2026**
**COI**	**None**

BMJ Open Review- BMJ Open 2026-121606

This protocol paper was well-written and key details were described in sufficient detail. My comments are minor and supportive of this work moving forward with publication.

1. Page 5: For an international audience, please define NHS the first time used.

2. Page 7: For an international audience, please orient reader to the West of Scotland Haven.

3. Page 7: Please clarify what the study's hypotheses are. Please state these clearly and connect with how methodologies will be used to test those hypotheses.

4. Page 9: Is delayed PGx the standard of care in Scotland? Please clarify. Is it possible that some of the locations covered by the study would involve locations where the standard of care is PGx testing? Would such affect the usual care comparison group? How do authors know that usual care group didn’t get PGx testing (contamination)? Please clarify.

5. Page 9: It is not clear how primary outcomes will be measured- how will the authors determine that ADR is related to index drug and not to other drugs/sources? How do authors define treatment failure? Please clarify how these outcomes were measured/defined.

6. Page 11: The authors do not cite a theoretical model for their work? Did they use any implementation science frameworks? Please clarify. It would strengthen their protocol if they did use such since it gives their work more rigor and structure.

7. Page 14: For inclusion/exclusion criteria, are there cases where index drug was prescribed but not picked up cancelled? If the focus of the protocol on those index medications prescribed and dispensed/picked up or only prescribed? If only prescribed and not necessarily dispensed, then the focus on prevention vs. intervention? Please clarify. Did they do an audit or review to see if prescribed index medications may have been canceled and affect implications of results?

8. Page 14: How did authors handle individuals who were on multiple index medications and to determine which one was the basis for inclusion or the focus of the study? The first one that appeared in database? Please clarify.

9. Page 18: Please clarify if the authors tracked those who were eligible but didn't enroll and reasons why they didn't enroll. It would be useful to determine recruitment bias.

10. Page 21: What is an actionable recommendation? Please define and provide examples.

11. Pages 21-22: How did the authors assess adherence to the index drug? If patient not adherent to index drug, the potential impact may not be as significant as the person who is adherence. If adherence measurement is not possible, please identify as a limitation of the methodological design. If adherence was assessed, please describe methodologies used.

12. Page 23: How do authors know the suspected ADRs are due to the PGx with the index drug? Please clarify. This may be a limitation of the study that should be noted.

13. Page 27: Why did the authors not consider performing both ITT and Per-protocol analyses? It might be useful to capture both analyses to determine impact of protocol adherence on study findings.

14. Page 27: Authors note logistical and linear regressions but do not state specifically their variables used in those analyses. It would be helpful to describe their variables and type of statistic so readers can assess the appropriateness of the statistical analyses.

15. Page 30-31: It is surprising the authors do not acknowledge limitations of their proposed design and how they will attempt to address these limitations. Such exploration would strengthen readers appreciation of the strengths and challenges of the protocol.

The authors are wished the very best as they seek publication of their protocol and conduct their study. It is hoped the authors get useful and impactful results.

VERSION 1 - AUTHOR RESPONSE

Reviewer 1

We thank the reviewer for a set of comments that have materially strengthened the protocol.

Comment 1. *For an international audience, please define NHS the first time used.*

Response:

The National Health Service (NHS) is now spelled out at first use and abbreviated thereafter.

Location in revised manuscript: *Introduction, Background and rationale, first sentence.*

Comment 2. *For an international audience, please orient the reader to the West of Scotland Safe Haven.*

Response:

We have added a definition of the West of Scotland Safe Haven at first use, describing it as an accredited Trusted Research Environment enabling secure, governance-approved linkage of routinely collected NHS data via the CHI number, without researchers accessing identifiable data directly.

Location in revised manuscript: *Methods, Study setting.*

Comment 3. *Please clarify the study's hypotheses and connect them with the methodologies used to test them.*

Response:

We have added an explicit Hypotheses subsection stating the primary and secondary hypotheses, each linked to its analytic method, while noting the pilot, estimation-focused framing.

Location in revised manuscript: *Introduction, Hypotheses subsection; methods detailed in Statistical analysis.*

Comment 4. *Is delayed PGx the standard of care in Scotland? Could some sites offer PGx as standard, affecting the comparison? How do you know the usual-care group did not receive PGx testing (contamination)?*

Response:

We have clarified that multi-gene panel PGx is not part of routine standard care at any participating site; current NHS Scotland practice is limited to a few nationally commissioned single gene-drug applications. We describe the safeguards against contamination: the assay is external and research-use-only, reports are never uploaded to the NHS record, and any routine single-gene test relates to a specific pathway captured in linked data. Routine single-gene testing during the trial will be monitored and reported.

Location in revised manuscript: *Methods, Interventions (comparator arm).*

Comment 5. *How will you determine that an ADR is related to the index drug and not to other sources? How is treatment failure defined?*

Response:

The primary-outcome definition now specifies blinded causality adjudication using validated tools (Liverpool Causality Assessment Tool or WHO-UMC), counting only events at least possibly related to the index drug, and provides a drug-class-specific operational definition of treatment failure with worked examples, with the full mapping pre-specified in the SAP.

Location in revised manuscript: *Methods, Outcomes (primary outcome).*

Comment 6. *The authors do not cite a theoretical model. Did they use any implementation science frameworks?*

Response:

We have added an Implementation framework subsection mapping the pre-specified implementation metrics onto Proctor et al.'s implementation-outcomes taxonomy and the RE-AIM framework, with references added.

Location in revised manuscript: *Methods, Implementation framework; references 18-19.*

Comment 7. *Is the focus on index medications prescribed and dispensed, or only prescribed? Did you audit whether prescribed index medications were cancelled?*

Response:

We have clarified that for inpatients an index drug requires an active HEPMA prescription that has proceeded to administration, and for outpatients that enrolment occurs only after the participant confirms they have received and commenced the medication (SA02); prescriptions cancelled before administration do not trigger enrolment.

Location in revised manuscript: *Methods, Eligibility criteria.*

Comment 8. *How were individuals on multiple index medications handled? Which drug was the basis for inclusion?*

Response:

Where more than one eligible index drug is newly prescribed during the same admission or eligibility window, the less frequently prescribed (rarer) drug is designated the primary index drug and determines stratification and the primary-outcome denominator. This prioritises representation of less common drug-gene pairs given the drug caps on high-frequency drugs. PGx recommendations in the intervention arm nonetheless apply to all index and concomitant medications.

Location in revised manuscript: *Methods, Eligibility criteria.*

Comment 9. *Did you track those eligible but not enrolled, and reasons why, to assess recruitment bias?*

Response:

Screening logs capture all individuals identified, approached, enrolled or not enrolled with reasons. We now state these will be summarised in a CONSORT-style flow diagram and used to characterise recruitment bias by comparing eligible non-participants with enrolled participants where governance permits.

Location in revised manuscript: *Methods, Screening and recruitment.*

Comment 10. *What is an actionable recommendation? Please define and provide examples.*

Response:

We have added a definition - a result that per CPIC/DPWG guidance warrants a change of drug or dose or added monitoring - with examples (CYP2C19/clopidogrel, CYP2D6/opioid), and defined a non-actionable result.

Location in revised manuscript: *Methods, Interventions (intervention arm).*

Comment 11. *How was adherence to the index drug assessed? If not possible, note as a limitation; if assessed, describe methods.*

Response:

Adherence is assessed at each monthly follow-up using a validated self-report measure, supplemented by CHI-linked community dispensing (proportion-of-days-covered), summarised descriptively by arm and incorporated into the CACE analysis. The limitations of self-report are acknowledged and mitigated by the linked dispensing data.

Location in revised manuscript: *Methods, Participant timeline and follow-up; Strengths and limitations.*

Comment 12. *How do you know suspected ADRs are due to the PGx interaction with the index drug? This may be a limitation.*

Response:

We have clarified that causal inference operates at two levels: event-level drug relatedness by blinded adjudication, and the genotype-mediated effect by the randomised comparison rather than individual-event adjudication. Residual attribution uncertainty is acknowledged as a limitation.

Location in revised manuscript: *Methods, Outcomes / Supplementary outcome-adjudication detail; Strengths and limitations.*

Comment 13. *Why not perform both ITT and per-protocol analyses?*

Response:

A per-protocol analysis has been added alongside the intention-to-treat primary analysis and the CACE analysis, with the per-protocol population defined by receipt of the allocated intervention as intended.

Location in revised manuscript: *Methods, Statistical analysis.*

Comment 14. *The specific variables in the regression models are not stated. Please describe the variables and statistics.*

Response:

For each model we now specify the outcome, covariates and effect measure: the primary logistic regression adjusts for the stratification factors plus age, sex, baseline polypharmacy count and SIMD quintile, reporting an adjusted odds ratio with 95% CI; secondary models and their effect measures are similarly specified, with full detail in the SAP.

Location in revised manuscript: *Methods, Statistical analysis.*

Comment 15. *The authors do not acknowledge limitations of the design and how these will be addressed.*

Response:

We have added a dedicated Strengths and Limitations section (in addition to the required box) covering the pilot scope and fixed end-date, the research-use-only nature of results, functional unblinding, ADR attribution uncertainty, self-reported adherence, limited ethnic diversity, and the external-laboratory model, with mitigations for each.

Location in revised manuscript: *Strengths and limitations (box after abstract; fuller discussion in the manuscript).*
