# Supplementary material for: PHOENIX: a study protocol for a pilot randomised controlled trial of pre-emptive pharmacogenomic panel testing in acute care settings with health economic evaluations
Source: BMJ Open. 2026 Oct 5;16(10):e121606. doi: 10.1136/bmjopen-2026-121606 (PMC13646959; doi:10.1136/bmjopen-2026-121606)
Supplement: Supplementary file 2 [file bmjopen-16-10-s001.docx]

# Supplementary MaterialsSupplementary Materials

**PHOENIX: A study protocol for a pilot randomised controlled trial of pharmacogenomic panel testing in acute secondary care with integrated health economic evaluations**

**Trial registration:** NCT06907784 | **Protocol version:** V3.2, 19 December 2025

This document contains supplementary tables, figures and methods referenced in the main manuscript.

# Contents:

- Supplementary Table S1 — Protocol amendment history
- Supplementary Table S2 — Full inclusion and exclusion criteria
- Supplementary Table S3 — Index drugs and associated pharmacogenes
- Supplementary Table S4 — Pharmacogenomic genes and variants
- Supplementary Table S5 — Schedule of enrolment, interventions and assessments (SPIRIT figure)
- Supplementary Methods S1 — Laboratory methods, PGx report generation, and action on receipt
- Supplementary Methods S2 — Baseline actionability and phenoconversion methodology
- Supplementary Methods S3 — Genotype quality control and reference-cohort benchmarking
- Supplementary Methods S4 - Outcome adjudication (supplementary detail)

## Supplementary Table S1: Protocol amendment history

| **Amendment Number**  **(protocol amendments only)** | **Date of Approval** | **Protocol Version** | **Rationale** |
| --- | --- | --- | --- |
| SA01 | 18 December 2025 | 2.0 | The originally approved protocol outlined a full RCT; however, due to a funding shortfall, we propose an amendment to reclassify the study as a pilot RCT with a reduced duration of 2 years (previously 3 years). Following expert consultation, it was agreed to retain the core RCT design while incorporating pilot objectives to assess feasibility, recruitment, and preliminary clinical and health-economic outcomes of pharmacogenomic (PGx) implementation in Scotland. Maintaining randomisation preserves methodological rigour and enables early efficacy assessment. The PHOENIX pilot trial will generate key preliminary data to inform future, fully powered studies and support wider PGx implementation in NHS. |
| NSA01 | 12 March 2025 | 2.1 | Following review, the Information Governance team requested the addition of statements to the participant information sheet to clarify how participant data will be used, ensuring that any actual or potential privacy risks are appropriately identified, assessed, and mitigated. We have also corrected minor typographical errors, including updating the follow-up duration to 3 months (previously stated as 6 months, consistent with SA01 approvals). Additionally, we have updated team member details in relevant sections of the protocol, tidied the amendment table, and made minor revisions to study posters. |
| NSA03 | 22 April 2025 | 2.2 | A small typo in the full PIS for Legal Representative (Welfare Attorney/Welfare Guardian/Nearest Relative) has been corrected, changing the follow-up duration from 6 months to 3 months. The protocol has been updated to include key trial contacts and the lay representative (TSC).  The original recruitment window required patients to be enrolled within 2 days of starting the index drug. This proved overly restrictive due to variability in dosing schedules across the 60 trial drugs. Extending the window to 3 days provides flexibility, accommodates weekends and public holidays, and maintains scientific and clinical relevance. The inclusion criteria (10.2.2, bullet point 3) now read: “Newly commenced in hospital on one of the drugs listed in the drugs eligible for inclusion in the trial. Consent should be obtained within 3 days of first index drug administration.”  Additionally, Table 17.1 (Schedule of Procedures for both A: Intervention Arm and B: Standard of Care Arm) has been corrected to remove surveys on medications and allergies/intolerances at Week 1. The Participant Perspective survey will now be sent at the End of Trial. |
| SA02 | 6 August 2025 | 3.0 | This amendment aims to enhance recruitment, refine outcome measures, and strengthen scientific rigour ahead of the trial’s end date (30th September 2026). Key updates include:  1. Recruitment and Sites  Expansion across NHS Greater Glasgow and Clyde (NHS GG&C) with additional hospitals and clinical departments.  Addition of NHS GG&C Mental Health Services as a new site, with Dr Maytal Wolfe as PI; Prof Sandosh Padmanabhan remains PI for other NHS GG&C locations.  Recruitment window extended from 2 to 3 days post-initiation of the index drug to improve eligibility capture while maintaining clinical and scientific validity.  2. Primary Outcome and PROMs  Primary outcome refined: “Incidence of drug-specific adverse drug events (ADEs) related to the index drug within three months of randomisation, comparing PGx-guided and standard care arms.”  PROMs tailored to reduce participant burden and capture drug-specific safety data via CASTOR eCRF, with monthly administration for 3 months.  Baseline data on pain, mental health, seizures, and hearing loss collected to support endpoint adjudication.  3. Safety and ADE Adjudication  Dual-pathway ADE adjudication introduced: ADEs contributing to the primary outcome vs serious/unexpected events requiring TSC oversight.  Clarification of SAE reporting, causality assessment, and safety monitoring responsibilities.  4. Inclusion/Exclusion Criteria  Minor clarifications and reformatting of tables for inpatient and outpatient settings.  New exclusion criteria: allogeneic haematopoietic stem cell transplant recipients, dialysis patients, and patients in other trials mandating index drugs.  Specific eGFR guidance for renal transplant patients on tacrolimus updated.  5. Participant Materials and Accessibility  PIS and consent forms revised for clarity, consistency, and accessibility, with separate inpatient/outpatient versions.  Minor typographical corrections (e.g., follow-up duration changed from 6 to 3 months).  Telephone withdrawal option added; participant and GP correspondence updated for medication changes.  NHS GGC “Clear to All” policy implemented for large-print materials.  6. Protocol and Operational Updates  PGx report review process clarified; additional drugs commenced before PGx report return will have recommendations communicated to the treating team.  Terminology standardised (“clinical research team”), trial website launched (<https://phoenix.gla.ac.uk>).  Statistical Analysis Plan (SAP) to be approved by TSC before database lock/unblinding; baseline comorbidity data updated per Charlson and Elixhauser indices.  DMC role removed, responsibilities assigned to TSC.. |
| NSA05 | 19 September 2025 | 3.1 | A typographical error in the exclusion criteria has been corrected: “estimated glomerular rate greater than 15 mL/min/1.73 m²” has been updated to reflect the original Protocol V2.2 wording, “estimated glomerular rate less than 15 mL/min/1.73 m²”  Tramadol has been added to the drug list on page 30; no study procedures are affected, as tramadol was already an index drug in PHOENIX. In the Standard of Care table on page 50, the “Possible change of medication” at 3 months has been corrected with an “X” to reflect a previous omission. |
| NSA07 | 19 December 2025 | 3.2 | The inclusion/exclusion criteria for index drug treatment duration have been revised: the total planned treatment length is now less than five consecutive days (previously less than seven days) to align with typical clinical practice for short-course antibiotics. The PHOENIX genetics lab, Gene by Gene, has upgraded from Veridose 1 to Veridose 2, enabling recruitment of patients prescribed flucloxacillin, which was previously paused due to Veridose 1 limitations. This update ensures trial eligibility reflects real-world prescribing standards and supports recruitment of antibiotic-treated patients. Additional protocol updates include clarification of information following the inclusion of NHS Golden Jubilee as a site, the addition of Oral Medicine, and improved description of the recruitment pathway. |

## Supplementary Table S2: (A) Inclusion Criteria and (B) Exclusion Criteria for Inpatients and OutpatientsSupplementary Table S2: (A) Inclusion Criteria and (B) Exclusion Criteria for Inpatients and Outpatients

### 2A: Inclusion criteria

| Inpatients | Outpatients (SA02) |
| --- | --- |

| - Age ≥18 years - Capable of giving informed consent directly or via a legal representative (e.g., next of kin, welfare guardian, health care power of attorney). - Participants who are newly prescribed one of the trial-eligible index drugs during their hospital stay may be approached for consent. Consent should be obtained within 3 days of the first dose of the index drug being administered.^NSA03^ If there is a clear clinical plan documented on HEPMA indicating that the patient will be started on an eligible drug (but has not yet received the first dose), consent may be obtained in anticipation. However, formal trial enrolment will only occur once the first dose of the index drug has been administered. - Participant must not have a prescription for this drug in the previous 3 months. - Participant is able to provide a cheek swab - Participant is able to take part and be followed-up for at least 12 weeks. - Participant is resident in NHSGGC health board area | - Age ≥18 years - Capable of giving informed consent directly or via a legal representative (e.g., next of kin, welfare guardian, health care power of attorney). - Participants who are expected to be prescribed a trial-eligible drug during their outpatient clinic visit may be approached for consent prior to starting the medication. In these cases, formal trial enrolment will only occur once the patient has confirmed that they have received and started the prescribed medication - Participant must not have a prescription for this drug in the previous 3 months. - Participant is able to provide a cheek swab - Participant is able to take part and be followed-up for at least 12 weeks. - Participant is resident in NHSGGC health board area. |
| --- | --- |

### 2B: Exclusion criteria

| Inpatients | Outpatients (SA02) |
| --- | --- |
| - Inability to give informed consent directly or via a legal representative. - Non-English speakers without translation support. - Participants co-enrolled in other trials where a medication is one of the index drug is part of the trial protocol. - Life expectancy estimated to be less than 6 months by treating clinical team. - Severe illness limiting participation (investigator discretion). - Duration of index drug total treatment length is planned to be less than five consecutive days.^NSA07^ - Not registered with a General Practitioner. - No fixed address. - Participant is, in the opinion of the Investigator, not suitable to participate in the trial. - Participant has existing impaired hepatic or renal function for which a lower dose or alternate drug selection is already part of current routine care. - Estimated glomerular filtration rate less than 15 ml/min/1.73m2 (except for participants with a renal transplant commenced on tacrolimus, who may be included regardless of eGFR, provided they are not on dialysis).eGFR result obtained at screening or within the last 6 months or patients record confirming history of CKD 5. ^SA02^ - Participants on any form of dialysis. ^SA02^ - Participant with advanced liver failure (stage Child-Pugh C). - Participants with liver transplant. ^SA01^ - Participants with allogeneic haematopoietic stem cell transplant. ^SA02^ - Participants previously enrolled in the PHOENIX trial. - Participant who has declined participation and has declined reapproach for subsequent drugs.   Index drug exceeding trial drug cap. | - Inability to give informed consent directly or via a legal representative. - Non-English speakers without translation support. - Participants co-enrolled in other trials where a medication is one of the index drug is part of the trial protocol. - Life expectancy estimated to be less than 6 months by treating clinical team. - Severe illness limiting participation (investigator discretion). - Duration of index drug total treatment length is planned to be less than five consecutive days. ^NSA07^ - Not registered with a General Practitioner. - No fixed address. - Participant is, in the opinion of the Investigator, not suitable to participate in the trial. - Participant has existing impaired hepatic or renal function for which a lower dose or alternate drug selection is already part of current routine care. - Estimated glomerular filtration rate less than 15 ml/min/1.73m2 (except for participants with a renal transplant commenced on tacrolimus, who may be included regardless of eGFR, provided they are not on dialysis)..eGFR result obtained at screening or within the last 6 months or patients record confirming history of CKD 5. ^SA02^ - Participants on any form of dialysis. ^SA02^ - Participant with advanced liver failure (stage Child-Pugh C). - Participants with liver transplant. ^SA01^ - Participants with allogeneic haematopoietic stem cell transplant. ^SA02^ - Participants previously enrolled in the PHOENIX trial. - Participant who has declined participation and has declined reapproach for subsequent drugs. - Index drug exceeding trial drug cap. |

Amendment references (SA01, SA02, NSA03, NSA07) correspond to Supplementary Table S1.

## Supplementary Table S3: Drugs with Pharmacogenomic Implications

| Medication | Gene 1 | Gene 2 | Gene 3 |
| --- | --- | --- | --- |
| Abacavir | *HLA-B* |  |  |
| Acenocoumarol | *CYP2C9* | *CYP4F2* | *VKORC1* |
| Allopurinol | *HLA-B* | *ABCG2* |  |
| Amitriptyline | *CYP2C19* | *CYP2D6* |  |
| Aripiprazole | *CYP2D6* |  |  |
| Atazanavir | *UGT1A1* |  |  |
| Atomoxetine | *CYP2D6* |  |  |
| Atorvastatin | *SLCO1B1* |  |  |
| Azathioprine | *TPMT* | *NUDT15* |  |
| Brivaracetam | *CYP2C19* |  |  |
| Capecitabine | *DPYD* |  |  |
| Carbamazepine | *HLA-A* | *HLA-B* |  |
| Celecoxib | *CYP2C9* |  |  |
| Citalopram | *CYP2C19* |  |  |
| Clobazam | *CYP2C19* |  |  |
| Clomipramine | *CYP2C19* | *CYP2D6* |  |
| Clopidogrel | *CYP2C19* |  |  |
| Clozapine | *CYP2D6* |  |  |
| Codeine | *CYP2D6* |  |  |
| Doxepin | *CYP2C19* | *CYP2D6* |  |
| Efavirenz | *CYP2B6* |  |  |
| Eliglustat | *CYP2D6* |  |  |
| Escitalopram | *CYP2C19* |  |  |
| Flecainide | *CYP2D6* |  |  |
| Flucloxacillin | *HLA-B* |  |  |
| Flucytosine | *DPYD* |  |  |
| Fluorouracil | *DPYD* |  |  |
| Flurbiprofen | *CYP2C9* |  |  |
| Fluvastatin | *CYP2C9* | *SLCO1B1* |  |
| Fluvoxamine | *CYP2D6* |  |  |
| Fosphenytoin | *CYP2C9* | *HLA-B* |  |
| Haloperidol | *CYP2D6* |  |  |
| Ibuprofen | *CYP2C9* |  |  |
| Imipramine | *CYP2C19* | *CYP2D6* |  |
| Irinotecan | *UGT1A1* |  |  |
| Lamotrigine | *HLA-B* |  |  |
| Lansoprazole | *CYP2C19* |  |  |
| Meloxicam | *CYP2C9* |  |  |
| Mercaptopurine | *TPMT* | *NUDT15* |  |
| Metoclopramide | *CYP2D6* |  |  |
| Metoprolol | *CYP2D6* |  |  |
| Nortriptyline | *CYP2D6* |  |  |
| Omeprazole | *CYP2C19* |  |  |
| Ondansetron | *CYP2D6* |  |  |
| Oxcarbazepine | *HLA-B* |  |  |
| Pantoprazole | *CYP2C19* |  |  |
| Paroxetine | *CYP2D6* |  |  |
| Phenprocoumon | *VKORC1* |  |  |
| Phenytoin | *CYP2C9* | *HLA-B* |  |
| Pimozide | *CYP2D6* |  |  |
| Piroxicam | *CYP2C9* |  |  |
| Pitolisant | *CYP2D6* |  |  |
| Pravastatin | *SLCO1B1* |  |  |
| Propafenone | *CYP2D6* |  |  |
| Risperidone | *CYP2D6* |  |  |
| Rosuvastatin | *SLCO1B1* | *ABCG2* |  |
| Sertraline | *CYP2B6* | *CYP2C19* |  |
| Simvastatin | *SLCO1B1* |  |  |
| Siponimod | *CYP2C9* |  |  |
| Tacrolimus | *CYP3A5* |  |  |
| Tamoxifen | *CYP2D6* |  |  |
| Tamsulosin +CYP2D6 inhib | *CYP2D6* |  |  |
| Tegafur | *DPYD* |  |  |
| Tenoxicam | *CYP2C9* |  |  |
| Tetrabenazine | *CYP2D6* |  |  |
| Thioguanine | *TPMT* | *NUDT15* |  |
| Tolterodine | *CYP2D6* |  |  |
| Tramadol | *CYP2D6* |  |  |
| Trimipramine | *CYP2C19* | *CYP2D6* |  |
| Venlafaxine | *CYP2D6* |  |  |
| Voriconazole | *CYP2C19* |  |  |
| Vortioxetine | *CYP2D6* |  |  |
| Warfarin | *CYP2C9* | *CYP4F2* | *VKORC1* |
| Zuclopenthixol | *CYP2D6* |  |  |

## Supplementary Table S4: Pharmacogenomic Genes and Variants

| Gene | Variants |
| --- | --- |
| ***ABCG2*** | *rs2231142* |
| ***CYP1A2*** | **1C, *1F, *1K, *7, *11* |
| ***CYP2B6*** | **4 , *6, *9, *18* |
| ***CYP2C19*** | **2, *3, *4, *17 (*4.002), *5, *6, *7, *8, *9, *10, *35* |
| ***CYP2C9*** | **2, *3, *4, *5, *6, *8, *11, *12, *13, *15, *18* |
| ***CYP2D6*** | **2, *3, *4, *6, *7, *8, *9, *10, *11, *12, *14, *15, *17, *18, *19, *21, *29, *29, *30, *31,*40, *41, *42, *49, *53, *54, *56, *58, *59* |
| ***CYP3A5*** | **3, *6, *7* |
| ***CYP4F2*** | *“3* |
| ***DPYD*** | **2, *13, c.2846A>T, c.557A>G, HapB3; c.1129-5923C>G* |
| ***HLA-A*31:01*** | **31:01* (rs1061235) |
| ***HLA-B*15:02*** | **15:02* (rs144012689) |
| ***NUDT15*** | c.415C>T, *3 (*2), rs116855232, c.416G>A, *4, rs147390019, c.37_42delGGAGTC (c.50_55del, c.50_55dup), *9 (*2, *6) rs746071566, c.80_81insCGGG, *14, rs777311140 |
| ***SLCO1B1*** | **5* |
| ***TPMT*** | **2, *3A, *3B, *3C, *4, *11, *29, *42* |
| ***UGT1A1*** | *28 |
| ***VKORC1*** | *1639G>A, c.196G>A, c.106G>T* |
|  |  |
| **Notes:** |  |
| (1) In the absence of any other variant analysed in the test, a *1 allele is called.  (2) Genotypes are paired combinations of each gene's variants as per table above. In addition, for *CYP2D6*, there are also other combinations involving *CYP2D6* copy numbers.  (3) *HLA* and *HLA-B* method changed from 25 November 2025 to a direct detection method provided with Veridose 2 on the Agena Mass array. *HLA-B*57:01* and *HLA-B*58:01* were also added for drugs abacavir, flucloxacillin and allopurinol.  (4) *UGT1A1* was only used in Veridose 1.0 only. | |

## Supplementary Table S5: Schedule of enrolment, interventions and assessments

## Supplementary Methods S1: Laboratory methods, PGx report generation, and action on receipt

### Sample collection and genotyping

Buccal swab samples are collected at enrolment using protocol-specified collection kits (Puritan HydraFlock swab, Puritan Medical Products, Guilford, Maine, USA). Samples are packaged in accordance with international shipping standards and couriered three times weekly to Gene by Gene (Houston, Texas, USA), a clinical genomics laboratory accredited under CLIA and CAP. Genotyping uses the Agena Bioscience Veridose pharmacogenomics panel (version 1.0, transitioning to version 2.0 from 25 November 2025); CYP2D6 copy-number variation is assessed using the Agena Veridose CYP2D6 CNV panel (CNV 1.0 until 25 November 2025, then CNV 2.0) (Supplementary Table S1: NSA07), consistent with the gene and variant list in Protocol v3.2.

The UGT1A1 assay could not be incorporated into the Veridose 2.0 array in time due to technical difficulties integrating the *28 allele; a standalone test was considered but its turnaround would not meet study requirements, so UGT1A1 was not included from version 2.0. Two assay chemistries were therefore used across the recruitment period. Gene and variant content and certain calling methods differed between them—for example, direct detection of HLA-B*57:01 and HLA-B*58:01 was introduced with version 2.0, and UGT1A1 was assayed on version 1.0 only. Analyses involving genotype data will account for assay version where relevant, and per-gene call rates will be reported by chemistry as part of the genotyping quality-control summary. DNA is extracted, quality-checked and processed using validated laboratory procedures.

### PGx report generation

Genotype data are transferred securely to myDNA Life (Melbourne, Australia), where UKCA-marked class I medical-device software generates a structured report. Reports summarise detected genotypes/diplotypes, the corresponding metaboliser or phenotype status (CPIC-based phenotype translation for target genes), and associated prescribing recommendations based on CPIC[17] and DPWG[9] guidance, focused on the index drug and clinically relevant concomitant medications.

### Action on receipt and review of the PGx report

The report, accessed via the secure Nucleus portal, is typically available within 10 days. Upon receipt it is reviewed by the trial physician, who communicates findings to the clinical team via a paper copy only, with instructions not to scan or electronically disseminate the report to avoid inclusion in health records. The treating physician uses clinical judgement to decide whether to implement recommendations, recording the decision in the eCRF; any prescription change follows usual ward practice. For patients discharged before the report is available, the trial physician contacts the GP to recommend medication changes in line with standard care, without including genetic details, and both patient and GP receive written notification. If additional medications are initiated before the report is returned (or within 7 days, whichever is earlier), relevant recommendations are also communicated. Where necessary, multidisciplinary discussion guides decisions on continuing, stopping or switching medications. Three consultant clinical pharmacologists support interpretation.

If a result cannot be generated (sample failure, inadequate DNA, assay failure, or unresolved interpretation), the participant remains in the trial and is followed according to randomised allocation. A repeat swab may be requested where feasible within a clinically relevant timeframe. Failed or unavailable results are recorded in the eCRF with the reason where known and included in analyses under intention-to-treat.

## Supplementary Methods S2: Baseline actionability and phenoconversion methodology

### Baseline whole-prescription actionability

To characterise the pharmacogenomic burden of the study population, the frequency of actionable drug-gene interactions is quantified at baseline across each participant’s entire prescribed medication list at consent, rather than for the index drug alone. This whole-prescription perspective reflects the intervention design, in which the report returns guideline-linked recommendations for all current medications while the primary endpoint is measured on the single pre-specified index drug. Baseline actionability is summarised descriptively, pooled across the cohort, across pre-specified nested drug scopes: the PHOENIX index-drug list; the complete set of CPIC/DPWG guideline-supported drug-gene pairs; and the broader PharmGKB/ClinPGx curated set of pharmacogenomic drug-gene interactions not yet incorporated into formal guideline recommendations (reported to enable comparison with published in-silico estimates). These are descriptive baseline measures, not effectiveness comparisons.

### Phenoconversion and effective phenotype

Genotype-predicted metaboliser status may be modified by co-prescribed enzyme inhibitors or inducers and by lifestyle factors (notably smoking, a strong CYP1A2 inducer), such that a participant’s effective phenotype differs from that predicted by genotype alone—termed phenoconversion. For example, a CYP2D6 genotypic normal metaboliser taking a strong CYP2D6 inhibitor may behave as a poor metaboliser.

PGx reports issued during the trial are based on genotype-predicted phenotype and do not incorporate concurrent inhibitor or inducer exposure. This reflects the intervention as delivered: the trial evaluates the effect of returning a genotype-based report, not a fully phenotype-adjusted recommendation. Separately, and for descriptive purposes only, predicted effective phenotypes are derived for every participant across each drug on their prescribed medication list at consent, by applying a locked, version-controlled rule set based on published inhibitor and inducer classifications together with lifestyle modifiers. Because the derivation uses only medications recorded at consent, effective phenotype is a baseline characteristic and is not updated using post-randomisation prescribing.

Phenoconversion is summarised descriptively at a series of thresholds of increasing stringency, from an inclusive definition counting modifiers of any strength to definitions restricted to strong modifiers. The clinically anchored measure counts only predicted shifts crossing the boundary between normal and non-normal metaboliser status, since it is at this boundary that a CPIC or DPWG dosing recommendation may change; this indicates the volume of clinical decision support that panel-based prescribing would generate in routine practice. All phenoconversion estimates are descriptive and pooled across the cohort, and phenoconversion does not enter the primary outcome analysis. Its role in exploratory subgroup and within-arm analyses is described in the main-text Statistical Analysis section.

## Supplementary Methods S3: Genotype quality control and reference-cohort benchmarking

As a quality-control step, observed allele and genotype frequencies for the panel markers are benchmarked against established European reference datasets (for example 1000 Genomes European, gnomAD non-Finnish European, UK Biobank European, PharmGKB, and published frequencies from previous European pharmacogenomic cohorts). Markers deviating materially from these references are subject to structured laboratory review and, where appropriate, orthogonal verification. This concordance assessment is a descriptive quality-control procedure independent of any trial outcome analysis. In a further descriptive comparison, predicted CPIC/DPWG phenotype frequencies for the major metaboliser genes are compared with corresponding published frequencies from previous European cohorts; close concordance would indicate that the trial cohort is genetically comparable to previously studied European populations but does not imply that clinical effects observed elsewhere would be reproduced in this setting.

## Supplementary Methods S4: Outcome adjudication (supplementary detail)

All suspected ADRs undergo blinded review by the clinical adjudication panel, which classifies each event using CTCAE v6 grading and applies causality assessment (Liverpool Causality Assessment Tool or WHO-UMC, by event type). Causal inference operates at two levels: whether an individual event is drug-related is determined by blinded adjudication; whether a genotype-mediated effect exists is inferred from the randomised comparison between arms and, within the intervention arm, from the comparison of outcomes between participants with actionable and non-actionable genotypes. It is the randomised design, rather than event-level adjudication, that supports causal interpretation of the pharmacogenomic effect.
